# Standardized Sample Preparation Using a Drop-on-Demand Printing Platform

**DOI:** 10.3390/s130505814

**Published:** 2013-05-07

**Authors:** Ellen L. Holthoff, Mikella E. Farrell, Paul M. Pellegrino

**Affiliations:** United States Army Research Laboratory, RDRL-SEE-E, 2800 Powder Mill Road, Adelphi, MD 20783, USA; E-Mails: mikella.e.farrell.civ@mail.mil (M.E.F.); paul.m.pellegrino.civ@mail.mil (P.M.P.)

**Keywords:** drop-on-demand, inkjet printing, sample preparation

## Abstract

Hazard detection systems must be evaluated with appropriate test material concentrations under controlled conditions in order to accurately identify and quantify unknown residues commonly utilized in theater. The existing assortment of hazard reference sample preparation methods/techniques presents a range of variability and reproducibility concerns, making it increasingly difficult to accurately assess optically- based detection technologies. To overcome these challenges, we examined the optimization, characterization, and calibration of microdroplets from a drop-on-demand microdispenser that has a proven capability for the preparation of energetic reference materials. Research presented herein focuses on the development of a simplistic instrument calibration technique and sample preparation protocol for explosive materials testing based on drop-on-demand technology. Droplet mass and reproducibility were measured using ultraviolet-visible (UV-Vis) absorption spectroscopy. The results presented here demonstrate the operational factors that influence droplet dispensing for specific materials (e.g., energetic and interferents). Understanding these parameters permits the determination of droplet and sample uniformity and reproducibility (typical R^2^ values of 0.991, relative standard deviation or RSD ≤ 5%), and thus the demonstrated maturation of a successful and robust methodology for energetic sample preparation.

## 1. Introduction

The development of systems capable of detecting and identifying explosive materials on surfaces at range is a priority for the United States Army. To properly assess detection and identification performance, emerging hazard detection systems must first be evaluated using calibrated samples under controlled conditions. Evaluation of systems based on optical detection techniques that allow for ranged sensing is complicated by spatial dependencies and a lack of a reliable means to generate calibrated reference samples containing the explosive materials. Therefore, standardized reference materials and preparation methods are needed for proper testing of system capabilities, the establishment of reliable benchmarks for system development, and to compare technology between systems. Furthermore, an assortment of explosive reference materials is needed to allow for flexibility in reacting to the diverse range of threats encountered [1]. A variety of techniques that offer temporary alternatives has been employed, including spray deposition [2] and drop-and-dry (dropcasting) methods; however, it is often observed that there is material waste or uneven sample coverage. In the latter case, material loading fluctuations over a given surface area may result in significant signal variance from laser-based detection systems that have beam diameters on a similar length scale. Recently, drop-on-demand inkjet printing technology has emerged as an effective approach to produce test materials to meet the requirements for sample standardization [3–7]. Unlike other sample preparation methods that often result in the “coffee ring” effect, for which most of the material is concentrated along the edges, samples prepared using drop-on-demand inkjet technology demonstrate excellent uniform material dispersion throughout [3,4,8–11]. The uniformity achieved using inkjet printing is based on the distribution of a multitude of microdroplets over a defined area (*i.e.*, an array of separated droplets). Although drop-on-demand inkjet printing may result in microdroplets that exhibit the “coffee ring” effect, this effect is statistically negligible as compared to drop-and-dry methods. This is of particular concern with spatially sensitive techniques such as optical interrogation.

Piezoelectric drop-on-demand inkjet printing is an efficient approach for the deposition of microdroplets of solutions onto a surface [12,13]. This technique is compatible with various solvents and substrates, providing precise control over material deposition. Additionally, a range of deposited material concentrations can be achieved by varying the number and spacing of microdrops printed. The inkjet fluid cannot be contaminated by the substrate or contamination on the substrate because it is a non-contact process, and thus the fluid can be easily dispensed into wells or other substrate features [13]. The reproducibility of optimized drop-on-demand systems has been reported to be better than 1% relative standard deviation (RSD) from measurement-to-measurement (within-day) and better than 2% RSD for day-to-day measurements of dispensed volumes [4,14]. These deviations are significantly lower than those observed for other sample preparation methods.

Recently, we have utilized a common commercial off-the-shelf (COTS) drop-on-demand printing platform for the preparation of a variety of samples to be used in both laboratory and field tests for the assessment of the hazard detection capability of an optical detection system at range. This drop-on-demand platform has not been specially modified, and therefore results and conclusions should be applicable for standard instrument users. Here, we report the development of this sample preparation method to produce both energetic and interferent test materials. It is not the intention of the authors to discuss the theory and basic principles of piezoelectric drop-on-demand inkjet printing (*i.e.*, fluid property, drive waveform, and orifice diameter effects) nor to discuss various applications for the technology, as this has been presented in numerous publications on the topic [12,13,15–24]. Instead, the purpose of this report is to describe basic methodology development, and provide an example of simple and inexpensive drop-on-demand system calibration measurements to verify the mass of various target materials deposited with each microdrop using a commercially available drop-on-demand system. Once the materials are deposited, the samples are used for hazard detection system evaluation and spectral data libraries. It is important to note that in some cases the polymorphic state of a material can be altered by the deposition process. This phenomenon has been discussed elsewhere as it applies to drop-on-demand inkjet printing and continues to be investigated by the authors [9,25,26].

## 2. Experimental Section

### 2.1. Reagents and Materials

Ammonium nitrate (AN), methanol (MeOH), distilled water (H_2_O), acetonitrile, sugar, urea, and potassium chlorate (KClO_3_) were obtained from Sigma-Aldrich (St. Louis, MO, USA). 1,3,5-Trinitro-1,3,5-triazine (RDX), octahydro-1,3,5,7-tetranitro-tetrazocine (HMX), 2,4,6-trinitrotoluene (TNT), and pentaerythritol tetranitrate (PETN) were obtained from Cerilliant (Round Rock, TX, USA). All inkjet printer stock solutions were prepared in a solution of methanol (MeOH) and water (v/v 2:1), acetonitrile (ACN) and water (20% by volume), or water, depending on solubility. Cerilliant standards were packaged in ACN. All stock solutions were sonicated for 30 min prior to use to ensure homogeneity. There was no need for filtering of the solutions to remove particulates before printing. All chemicals were used as received unless otherwise noted. Materials produced for validation and process reproducibility experiments in our laboratory were stored in a low temperature oven (30–40 °C).

### 2.2. Inkjet Printing

**Figure 1.**
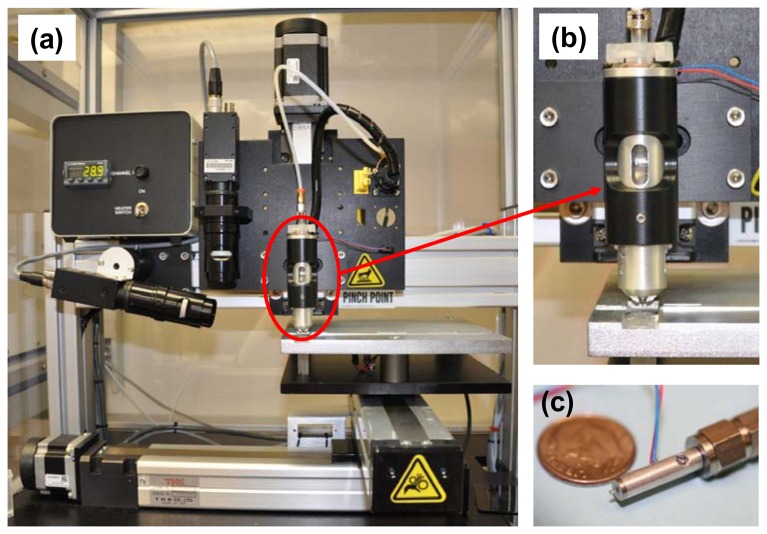
Photographs of (**a**) JetLab^®^ 4 drop-on-demand inkjet printing platform; (**b**) dispensing device and ink solution encasement; and (**c**) print head assembly.

Test materials were fabricated using a JetLab^®^ 4 (MicroFab Technologies, Plano, TX, USA) tabletop printing platform. The system is shown in Figure 1(a). The JetLab^®^ 4 is a drop-on-demand inkjet printing system with drop ejection drive electronics (JetDrive™ III), a drop visualization system, and precision *X*, *Y*, *Z* motion control. The standard manual pressure controller (Fairchild^®^, Winston-Salem, NC, USA) was upgraded with a COTS electronic pneumatic pressure controller (MicroFab). The dispensing device (print head assembly, MJ-AL-01-060) consists of a glass capillary tube with a 60 μm diameter orifice coupled to a piezoelectric element. Photographs of the dispensing device encasement and the print head assembly are shown in Figure 1(b,c), respectively. Voltage (V) pulses applied to the piezo result in pressure fluctuations around the capillary. These pressure oscillations propagate through the printing fluid in the tube, resulting in ejection of a microdrop. Both sinusoidal waveform voltage pulses and standard waveform bipolar voltage pulses were used to generate microdrops. The echo (negative) part of the bipolar pulse is used to cancel the residual acoustic waves propagating in the tube and therefore optimize the drop break-off.

Determining optimal jetting parameters requires trial-and-error. Stable droplet ejection is achieved by visually observing expelled microdrops and adjusting voltage pulse parameters and capillary fluid backfill pressure to create an “ideal” drop. Drops are visualized using synchronized strobe illumination and a charged coupled device (CCD) camera. Printing was performed at a frequency of 250 Hz with a droplet velocity of ∼2 m/s. Droplet velocities between 1.5 m/s and 2 m/s are ideal for printing. Droplet velocities less than 1 m/s may result in inaccurate drop placement [27]. Conditions that provide the highest drop velocity without satellite droplet formation are desired. Drop diameter was estimated to be ∼60 μm, based on the capillary orifice diameter. For clarification, the dwell time is the time during which the piezo wave form changes shape when a drive voltage is applied to the piezoelectric device for a given amount of time. An optimal drop is a droplet typically equal in size to the dispensing orifice being used, which does not have satellites, and consistently falls at an optimum velocity. Satellites are secondary droplets, ejected following the optimal droplet, typically observed to be smaller in volume than the optimal droplet. Satellites deposited add to the total concentration error and can affect droplet spacing uniformity.

During printing, a single substrate or vessel was placed on the sample stage. The print head remained fixed at a specified height while the stage moved to print a specified pattern or number of microdrops. A rectangular area, which covers a substrate region with rows and columns of equidistant points, was pre-programmed based on the vessel size. The total number of drops needed to achieve a desired concentration per unit area is calculated based on the volume of a single microdrop and the solution concentration. Based on the number of total drops needed, the array spacing and drops needed per line can be calculated. These values are easily adjusted depending on solution concentration. Patterns were printed using the print on-the-fly mode. In this mode, the stage moves continuously as a single microdrop is dispensed at each array element and is therefore governed by the microdroplet density and the velocity of the sample stage. Although print on-the-fly mode, which deposits droplets without stopping the translation stage, improves sample throughput, a limitation to using this mode is the ability to print samples with droplet spacing less than 70 μm.

### 2.3. UV-Vis Absorption Spectroscopy

UV-Vis absorption spectroscopy measurements were collected using a Shimadzu^®^ UV-3600 UV-VIS (Columbia, MD, USA) spectrophotometer. Calibration curves were constructed by measuring the absorbance spectra from standard solutions (of known sample concentrations) containing various pre-determined energetic and interferent compounds. The evaluated analytes were: AN, potassium chlorate, HMX, TNT, RDX, PETN, urea, and sugar. The analyte of interest was dissolved in the appropriate solvent and then diluted to various concentrations. Absorbance was measured using quartz cuvettes (1 cm path length) in a dual beam UV-Vis. One cuvette was filled with 3 mL of analyte solution (sample), and the other was filled with 3 mL of pure solvent (blank, H_2_O). Wavelength scans from 190–400 nm were used to measure the absorbance of the various target materials at known concentrations. The analytes of interest had absorption features in this wavelength region. Data analysis was completed using UV Probe software (version 1.10).

## 3. Results and Discussion

We report microdroplet optimization results for a 60 μm orifice dispensing device with analyte samples in conjunction with ACN, ACN and H_2_O (20% by volume), 2:1 MeOH-H_2_O, and H_2_O solvents. We also discuss the performance for the microdrop mass calibration method described, as applied to selected inkjet stock solutions. These results also include the determined repeatability and standard uncertainties of the experimental measurements.

### 3.1. Microdroplet Optimization

Prior to analyte deposition, it was important to have a working knowledge of how the drop-on-demand instrument settings, analyte concentration, and inkjet printing solvent viscosity (which can also be a function of analyte concentration), influence the character of droplets produced. Many variables impact the drop; therefore, optimized parameters are needed to achieve the best drop at a sufficient velocity. Controlling the physical properties (e.g., size, volume) of the dispensed microdrops will ultimately also affect droplet variation and reproducibility. The volume of the dispensed microdroplet is a function of the printing fluid, dispensing device orifice diameter, and waveform parameters. Based on these factors, the droplet volume can range from 50 to 200 pL [28]. In all cases, under the conditions tested it was found that in order to maintain a consistent drop with a high velocity, several variables needed to be optimized, including the pressure set point, dwell time (standard waveform), period (sinusoidal waveform), and voltage applied to the dispensing device. The pressure set point required minimal changes within-day and day-to-day. Variations in the fluid level inside the printing solution encasement required changes in the pressure set point [27]. The laboratory temperature and humidity varied from 21 °C to 23 °C and 16% to 20%, respectively. All analyte solutions were dispensed at room temperature. Although microscopic optical imaging was available on the COTS system to measure ejected droplet dimensions, it was not utilized due to large volume and mass uncertainties [29]. In general, H_2_O-containing solvents produced visually larger droplets (in flight). Larger droplets were also visualized when sinusoidal waveform parameters were used to generate microdroplets.

The parameters shown in Table 1 for the analytes of interest in suitable solvents were uniquely capable of producing consistent drops within-day and day-to-day (±3 μs and/or V) using standard waveform settings (*i.e.*, consistent drops were visualized at or close to the same dwell time and voltage settings each day). It is important to discuss some of these settings in relation to solvent and solution concentration. Table 1 is meant to be a starting point or guide for users employing similar instrumentation. Three different concentrations of AN were investigated in a 2:1 MeOH-H_2_O solvent. While AN is soluble in H_2_O alone, a 2:1 MeOH-H_2_O solvent (maintaining AN solubility) was also employed to increase the drop evaporation rate, and allow for the fabrication of neat high concentration test substrates (droplets printed remained in specific location, without concern for solution pooling). Furthermore, MeOH did not compromise substrate coatings (e.g., clear coat, paint). Generally, the concentration of AN did not significantly affect the settings for maintaining a consistent drop.

H_2_O (20% by volume) was added to the Cerilliant explosive standards (TNT, HMX, RDX, and PETN). This was done to minimize damage to substrate coatings from ACN. The parameters used for printing TNT, HMX, RDX, and PETN in the ACN-H_2_O solvent were comparable, which was expected based on similar solution concentrations and solvent characteristics. Additional optimization was completed for Cerilliant RDX standard in ACN solution, for analyte deposition onto uncoated substrates. In comparison to the ACN-H_2_O solvent, the parameters used for printing RDX in ACN are not drastically different; however, it was more difficult to achieve a consistent drop using an ACN solvent This may be due to solvent properties (e.g., viscosity). Furthermore, the lower voltage values may be related to a change in solvent viscosity, as decreasing viscosity causes a decrease in voltage required to eject a microdroplet [16].

We were limited to printing KClO_3_, sugar, and urea in H_2_O due to minimal solubility in the other solvents of interest investigated. For sugar and urea, the increased viscosity of H_2_O, in comparison to the other solvents investigated, resulted in an increase in the voltage required to create a drop. KClO_3_ did not require higher voltages for microdroplet formation. This may be because of the KClO_3_ molecules dissolving and then breaking into potassium and chlorate ions in H_2_O. An additional concern was the interaction of adjacent deposited droplets (*i.e.*, multiple droplets combining or pooling); however it was observed that an increase in voltage decreases the effective orifice diameter of the dispensing device, thus decreasing the size of the microdroplet [16]. In summary, droplet interaction was not observed for any of the investigated analyte solutions.

The JetDrive™ III drop ejection drive electronics also operated in a sinusoidal waveform mode. This waveform was initially investigated in an effort to increase analyte mass deposited (as compared to using the standard waveform), and possibly improve RSD values. The sinusoidal waveform was investigated for several analytes, example parameters are explained below for the Cerilliant RDX standard. The parameters shown in Table 2 were uniquely capable of producing consistent drops within-day and day-to-day (±2 V and/or μs) using sinusoidal waveform settings. In order to maximize analyte concentration and therefore analyte mass deposited with a microdroplet, H_2_O was not added to ACN.

### 3.2. Microdroplet Mass Determination

**Figure 2.**
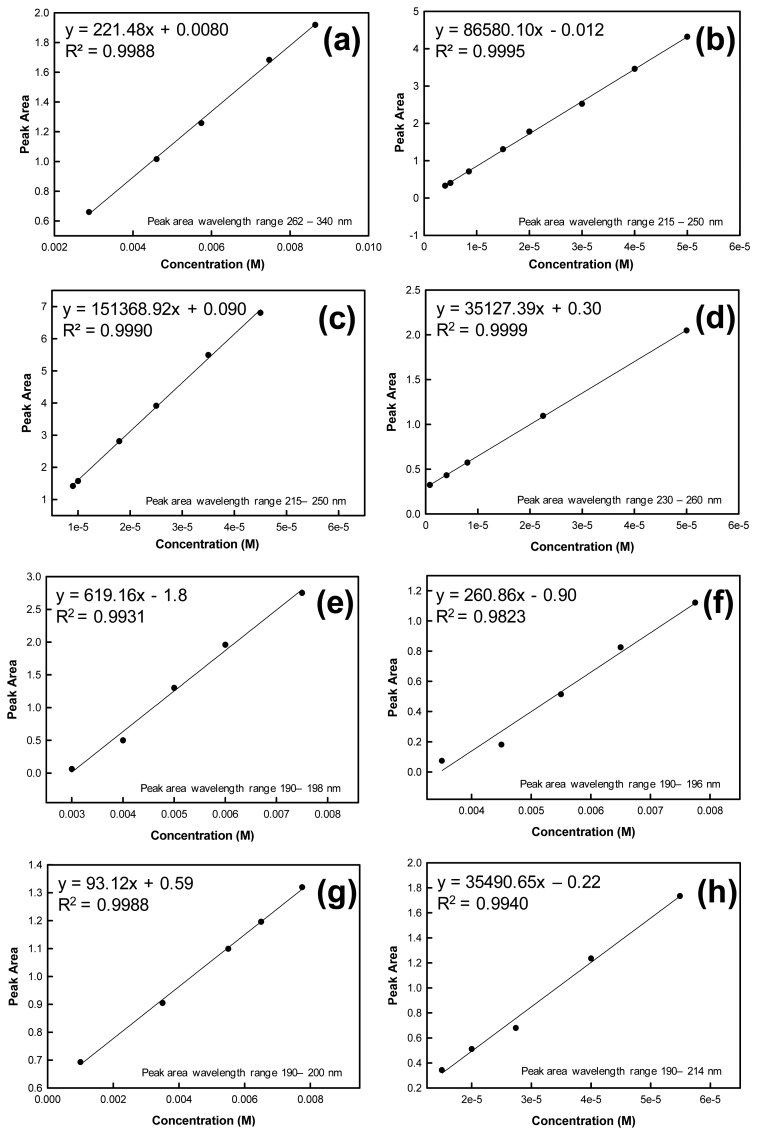
Calibration curve and R^2^ value from one UV-Vis data set at various analyte concentrations for (**a**) AN; (**b**) TNT; (**c**) HMX; (**d**) RDX; (**e**) urea; (**f**) potassium chloride; (**g**) sugar; and (**h**) PETN. Wavelength ranges used to determine the peak areas for each analyte are also given.

To determine the mass of analyte contained in each ejected microdroplet, absorption spectra were recorded for known concentrations of the analyte. A calibration curve was constructed using these concentrations and absorbance spectral peak areas calculated by the instrument software and based on the specified wavelength range. Calibration curves were used to prepare linear regressions from which the microdroplet mass and standard error could be calculated using the equation of the line and goodness of fit value (R^2^ value). The solutions used to construct the calibration curves were independent from the solutions used in the drop-on-demand inkjet printer for droplet mass determinations. Calibration curves for the evaluated analytes are shown in Figure 2.

The mass of each analyte of interest deposited per drop was determined by dispensing known numbers of drops into a Petri dish containing a known volume of solvent. Spectral peak areas for these droplets in solution were determined from the UV-Vis absorbance spectra. These y values were then substituted into the respective calibration curve equation (y = mx + b) to determine solution concentration.

Based on this concentration, volume of solvent and total number of drops dispensed, the mass of a single droplet could be calculated. For example, considering the RDX peak area from 230 nm to 260 nm, the linear fit equation for the RDX calibration curve is y = 35127.39x – 0.30. Based on the peak area for an unknown printed sample of RDX (2 arrays printed into 3 mL of H_2_O, 2.80 × 10^4^ drops per array), the predicted concentration from the calibration curve equation is 2.18 × 10^−5^ M RDX. The following calculations were completed to determine the mass of RDX in a single microdrop:

2.18 × 10^−5^ mol/L × 0.003 L = 6.54 × 10^−8^ mol
6.54 × 10^−8^ mol × 222.12 g/mol = 1.45 × 10^−5^ g RDX (total mass)
1.45 × 10^−5^ g TNT/56,000 drops = 2.59 × 10^−10^ g RDX/drop = 2.59 × 10^−4^ μg RDX/drop

It is important to note that if the total number of drops dispensed exceeds 1.0 × 10^5^, the added volume may need to be considered when calculating the mass of a single microdroplet. Masses were calculated in μg as test sample concentrations are calculated and prepared in μg/cm^2^.

This methodology was repeated at least three times per run, and resulted in excellent RSD values of ≤5% using the bipolar waveform (e.g., 2.7% and 3.9% RSD for AN and TNT, respectively) and ≤10% using the sinusoidal waveform (e.g., 7% RSD for RDX). In general, better RSD values were achieved for samples printed in ACN-H_2_O as opposed to ACN only. As stated above, this may be related to solvent properties (e.g., viscosity). RSD values ≤10% were sufficient for the preparation of samples and calibration was not repeated over the course of a typical sample set preparation However, calibration was repeated before starting a new sample set. RSD values for long term (*i.e.*, months) stability of microdroplet mass were typically ≤15%. Microdrop mass averages and RSD values were determined by averaging at least three successive additions of a single array to a known solvent volume. These values are provided in Table 3 for the energetic analytes of interest. Based on these results for RDX using different solvents and waveforms, three different microdroplet mass values were obtained. The sinusoidal waveform parameters produced a larger droplet mass compared to the bipolar waveform parameters using both the ACN-H_2_O solvent and ACN only; however, there was no significant (*i.e.*, orders of magnitude) increase in overall deposited analyte mass. Similar results for TNT, HMX, and PETN Cerilliant standards were determined (data not shown).

### 3.3. Printing Parameters

By determining the mass of material dispensed per droplet, test materials containing a known range of sample concentrations were successfully produced. Based on the desired sample area and analyte concentration within that area (μg/cm^2^) to be printed, the total number of microdroplets needed to cover the area and the droplet spacing could be calculated. The following calculations were completed to determine these printing parameters for a 25 μg/cm^2^ sample of RDX having a sample area of 25.80 cm^2^ (2,580 mm^2^):

(5 μg/cm^2^ × 25.8 cm^2^)/(4.30 × 10^−4^ μg RDX/drop) = 3.00 × 10^5^ total drops needed
(2,580 mm^2^/3.00 × 10^5^ total drops)^½^ = 0.0927 mm spacing between drops
50.8 mm sample length/0.0927 mm spacing = 548 drops per row (lengthwise, y)
50.8 mm sample width/0.0927 mm spacing = 548 drops per row (widthwise, x)

The spacing and number of drops per row in each direction were used with the drop-on-demand inkjet printer software to print an array. Similar calculations were made to determine microdroplet spacing and drops per row for additional areal concentrations and the other analytes of interest discussed here (results not shown). In order to maintain separation of droplets on the sample substrate, the space between droplets is limited by the solution behavior (e.g., evaporation rate, surface tension) once a microdroplet is deposited onto the substrate. This spacing limitation is important in relation to the type of optical detection technology being assessed using this sample preparation method. The dimensions of the interrogating beam area must be considered to ensure the areal concentration is uniform.

### 3.4. Method Validation

The preparation of standardized samples requires a microdroplet mass calibration method to ensure sample reproducibility and uniformity. Additionally, a secondary method should be employed to validate microdroplet mass determinations. The calibration method discussed in this report was verified using a sensitive microbalance (Mettler-Toledo XP2U, Columbus, OH, USA) (data not shown) [3]. Further method validation was completed by an external laboratory using ion chromatography (data not shown). It is also important to note that although spectroscopy techniques offer a simple and viable calibration method, no single standalone technique is appropriate for the plethora of chemical hazards that exist. For example, UV-Vis absorbance spectroscopy, which worked well for the majority of analytes of interest discussed here, may prove difficult for some materials that do not exhibit strong absorbance features in the wavelength range of the instrument. The use of a gravimetric method, such as a sensitive microbalance, is a practical alternative, as excellent precision (<2% RSD) in the determination of droplet mass can be achieved using appropriate experimental considerations [29]. Furthermore, COTS drop-on-demand inkjet systems are available with an integrated microbalance.

## 4. Conclusions

Our results demonstrate a sample preparation protocol that produces uniform samples to be used for utility assessments of emerging optical hazard detection technologies. This method for determining the mass of material contained in droplets ejected from a drop-on-demand inkjet printer allows for system calibration and the preparation of specific sample material mass loadings. Optimization of microdroplet formation provides microdispensing with specific drop placement and pattern printing capabilities. Based this report, we consider the use of a COTS drop-on-demand system in combination with our analysis and validation techniques to be a reasonable method for the fabrication of standardized reference energetic and interferent test materials, and should be considered a predominant and universal innovative industry standard. However, further investigations into analyte polymorphism and long-term sample stability are needed.

## Figures and Tables

**Table 1.** Standard waveform parameters for an optimal drop using ACN, ACN and 20% H_2_O (by volume), H_2_O, and a 2:1 MeOH:H_2_O ratio solution with a 60 μm dispensing device orifice.

**Analyte**	**Solvent**	**Solution Concentration (M)**	**Dwell Time (μs)**	**Echo Dwell Time (μs)**	**Voltage (V)**	**Echo Voltage (V)**	**Pressure Set Point (psi)**
AN	MeOH-H_2_O	1.2	30	34	18	−19	−0.070
AN	MeOH-H_2_O	0.10	27	32	16	−17	−0.070
AN	MeOH-H_2_O	0.010	28	33	17	−16	−0.051
TNT	ACN-H_2_O	0.037	31	36	18	−17	−0.068
HMX	ACN-H_2_O	0.027	32	31	21	−18	−0.028
RDX	ACN-H_2_O	0.038	29	38	18	−17	−0.070
PETN	ACN-H_2_O	0.026	31	36	18	−16	−0.050
RDX	ACN	0.045	33	39	14	−13	−0.060
KClO_3_	H_2_O	0.52	32	32	14	−18	−0.040
Sugar	H_2_O	0.75	35	35	25	−25	−0.030
Urea	H_2_O	0.99	35	36	24	−23	−0.042

**Table 2.** Sinusoidal waveform parameters for an optimal RDX drop using ACN with a 60 μm dispensing device orifice.

**Analyte**	**Solvent**	**Solution Concentration (M)**	**Initial Voltage (V)**	**Peak Voltage (V)**	**Period (μs)**	**Pressure Set Point (psi)**
RDX	ACN	0.045	0.0	40.0	100.0	−0.055

**Table 3.** Inkjet target analyte solution concentrations and corresponding calibrated droplet mass.

**Analyte**	**Solvent**	**Solution Concentration (M)**	**Analyte Mass Per Single Drop (μg)**	**Waveform**
AN	MeOH-H_2_O	1.2	1.15E–02	bipolar
AN	MeOH-H_2_O	0.10	8.31E–04	bipolar
AN	MeOH-H_2_O	0.010	4.24E–05	bipolar
TNT	ACN-H_2_O	0.037	7.96E–04	bipolar
HMX	ACN-H_2_O	0.027	5.01E–04	bipolar
RDX	ACN-H_2_O	0.038	4.30E–04	bipolar
PETN	ACN-H_2_O	0.026	7.72E–04	bipolar
RDX	ACN	0.045	2.51E–04	bipolar
KClO_3_	H_2_O	0.52	4.11E–03	bipolar
Sugar	H_2_O	0.75	1.98E–02	bipolar
Urea	H_2_O	0.99	6.44E–03	bipolar
RDX	ACN	0.045	8.87E–04	sine
